# Physiological Pacing: Historical Review With an Eye to the Future

**DOI:** 10.1111/jce.16559

**Published:** 2025-01-07

**Authors:** Richard Sutton, Atul Prakash

**Affiliations:** ^1^ Department of Cardiology, Hammersmith Hospital Campus National Heart & Lung Institute, Imperial College London UK; ^2^ Department of Cardiology, St Mary's Hospital Rutgers's University New Brunswick New Jersey USA

**Keywords:** diastolic function, physiological pacing, stimulus delivery, stimulus timing, systolic function

## Abstract

This review provides a history of physiological pacing from inception to current practice and into the future. This review stems from personal experience and is not formally systematic. Physiological cardiac pacing is covered from 1960s to date. Concepts, and major milestones with their practical applications are reviewed including possible applications in the future. Huge strides have been made in the last 50 years, but consequences of developments have not always been well considered resulting in important adverse effects. The future requires deep electrophysiological thinking to achieve further benefits for our patients.

## 1 Introduction

The concept of physiological pacing is ever evolving as we increasingly understand the close relationship of electrical activation and mechanical function. We also comprehend that this is different in normal and diseased hearts. Undoubtedly, in comparison with a normal heart having a normal electrical system, the best one can do to restore physiology is to simulate the normal electrical system which may be expressed by the phrase–“One cannot beat nature.” In a diseased heart, physiological pacing should attempt to alter the electrical system to achieve an optimal electromechanical state. Hitherto, our focus has been largely on ventricular systolic function, its preservation and improvement. This may be only one part of the journey which must include both ventricular diastolic and atrial function and timing; throughout the modern period we have been naïve in our understanding of the intricate relationship between these aspects of overall cardiac function. In this review, we offer a broad view of physiological pacing from its beginnings to where we are now, plus a glimpse of the future. When we look back, we see that each idea or concept as logical, as it seems now, and took 10–15 years for the pacing world to incorporate.

## 2 Early Attempts at Physiological Pacing

Physiological pacing, it may be said, began in the 1970s with the first and second publications, using this as title of a publication, reported re‐coordination of atrial and ventricular contraction. In this process, either a single lead carrying atrial and ventricular electrodes [1] or atrial and ventricular leads with dual sensing and triggered ventricular pacing was used [2]. The first clinical devices for transvenous pacing were VAT [3], atrial sensing ventricular triggering or, later, VDD, atrial sensing atrial triggered ventricular pacing (on‐demand). Before this, there had been dual chamber pacing systems with epicardial leads even in the 1960s, but they had not been successful due to high current drain and inadequate batteries to match this demand [4]. The very first step in the 1960s was VVI or ventricular demand pacing (also VVT but this mode never caught on). The idea hinged on sensing spontaneous ventricular depolarization through the pacing lead which delayed a further ventricular stimulus by restarting the R‐R' interval of the device. The effect avoided R on T stimulation. The concept was invented more or less simultaneously by Berkovits in United States and Davies in United Kingdom in the mid‐1960s resulting in a bitter patent legal battle which Berkovits won [5, 6].

The first truly physiological devices were VAT (full descriptions of pacing modes are available [3]) soon becoming VDD which delivered what was then desired, atrioventricular (AV) synchrony. Berkovits, in the late 1960s, made AV sequential pacing possible but this device lacked atrial triggering of ventricular stimulation, having only DVI as a physiological mode [7]. The VAT/VDD devices commonly presented pacemaker mediated tachycardia (PMT) because the atrial refractory period was too short. However, the VDD devices were sufficiently good to be those used in the first randomized controlled trial (RCT) in pacing. A comparison was made between VDD and VVI modes [8] in a small group of 13 patients. Significant benefits were shown for VDD over VVI mode in terms of symptoms (dyspnea and wellbeing) and exercise capacity. At this time, RCTs were relatively new and underexplored. A device trial raised new difficulties compared with drug trials which had begun years earlier. Much was learnt about device trial design over subsequent years. It was soon clear that this early trial included too few patients to have sufficient impact to change pacemaker policy. Twenty years later, much larger, better designed trials were conducted [9, 10, 11]. Nevertheless, these trials were completed in parallel with established clinical behavior, widespread use of dual chamber pacing in the majority of patients requiring pacing, except those in persistent/permanent atrial fibrillation (AF). For these AF patients and others rate‐responsive ventricular pacing (VVIR) was introduced in the mid 1980s on a realistic basis with activity sensing engineered to yield a heart rate increase in parallel with increasing activity [12]. The later trials only provided confirmation for the already established dominant change from single chamber ventricular pacing to dual chamber. It was as if all the pacemaker doctors knew that dual‐chamber pacing was better even if the trials showed only limited benefit. Administrators/budgeteers did not want physiological pacing, but they failed to squeeze it out despite the lack of overwhelming trial evidence.

The 1980s added the so‐called universal pacemaker of Funke [13], which included atrial pacing in addition to VDD, termed DDD. It had a longer atrial refractory period largely eliminating PMTs. It slowly became the norm of physiological pacing with all manufacturers offering similar devices. The trials which followed [9, 10, 11] showed some benefit of physiological pacing, but it was not what the profession expected and, although the trials themselves were very diligent, it seems fair to say that they were not of great influence.

Rate responsive pacing (AAIR, VVIR, DDDR [3]), which is now beyond debate, also had a significant role in the evolution of physiological pacing with a simple aim of providing patients with rising heart rate on exercise. There have been numerous algorithms tested including sensing of activity, minute ventilation, and more recently right ventricular impedance, reflecting both volume and contractility in ‘Closed loop stimulation’. Sensing function has extended beyond detecting need for rate increase to roles in vasovagal syncope and atrial fibrillation [14, 15].

We had, at this stage, learned much but there was and is so much more to comprehend. It is necessary to pursue physiological pacing in its totality, and by so doing we may avoid some pitfalls such as we have already faced on this journey.

## 3 Evolution of Physiological Pacing: Atrial Pacing

In the early days of physiological pacing, the operators found that securing a stable electrode position in the right atrium was a considerable challenge. As dedicated equipment was made available and skills improved the challenge was overcome, mainly with active fixation leads and, in some instances, catheter delivered leads. However, with atrial pacing little thought was devoted to atrial stimulation site, the choice was right atrial appendage because that site was where leads were stable. Even today, when a lead can be placed anywhere, insufficient speculation on the non‐physiological site that is the right atrial appendage. Nevertheless, the first DANPACE study [16] was very influential in pacing for sinus node disease (SND). It was shown that atrial pacing was all that was needed in many cases of SND, and, in their series, there was a very low incidence of development of AV block. However, others found a low but unacceptable incidence of ventricular conduction delay [17]. The second DANPACE trial [18] showed a greater occurrence of atrial fibrillation with AAIR than in DDDR pacing in SND which was attributed to long effective AV intervals with AAIR mode. No comment was made on the possibility of the atrial stimulation site being at fault was included. The outcome was that there was a very small uptake of atrial pacing (AAI/AAIR) internationally.

## 4 Atrial Activation and Function: An Unrecognized Component of Physiological Pacing

Despite the ongoing advances in addressing normalization of ventricular activation and its relationship to systolic and diastolic function, atrial function with pacing is still being overlooked. Pacing from the right atrial appendage, even at its root, prolongs P wave duration and alters activation patterns in the lateral right atrium and left atrium [19]. As in the ventricle, this abnormal activation has been shown to be arrhythmogenic [20]. By way of illustration, some patients with paroxysmal atrial fibrillation and heart failure with preserved ejection fraction, when in sinus rhythm have prolonged P waves and atrial dyssynchrony has been demonstrated by echocardiography [21]. Considerations similar to those in ventricular pacing need to be directed to the atria, including how to take advantage of Bachmann's bundle [22], and the possibility of dual site atrial pacing [23]. The atria unlike the ventricles do not have a well‐defined specialized conduction system, though the debate continues. However, in patients with significant inter‐ and intra‐atrial conduction delay, pacing from different sites within the right atrium can significantly shorten activation times in diseased atria [24, 25]. This can translate into significantly improved atrial function and ventricular filling [26, 27]. Pacing from the interatrial septum, the coronary sinus and Bachmann's bundle can also pre‐excite the left atrium in some disease states [24]. Pre‐excitation of the left atrium with dual site atrial pacing has been shown to reduce the recurrence of atrial fibrillation (AF) on a background of either antiarrhythmic drug therapy and/or ablation [23]. Nagarakanti et al. have shown that dual site atrial pacing can achieve long‐term atrial reverse remodelling and preserved AV systolic function. This may contribute to long‐term rhythm control in patients with atrial fibrillation refractory to antiarrhythmic drugs or catheter ablation [28].

## 5 Evolution of Physiological Pacing: Ventricular Pacing

The electrical conduction system of the ventricles, and their mechanical function are entwined. Despite being vividly described by Wiggers in the 1920s [29], the relationship between the two has only become slowly but increasingly recognized. However, even now minor changes in electrical patterns and their impact on cardiac structure and function remains incompletely understood or appreciated. Undoubtedly, the native normal electrical system is the best for function of the normal heart. With the understanding that true physiological pacing needs to reproduce the intrinsic ventricular activation, it is essential to understand ventricular activation patterns and the mechanical architecture of the two ventricles [30]. When abnormalities emerge with disease and aging, intervention is instituted to, at best nearly, normalize the electrical patterns and, thus, function. A good example is the use of cardiac resynchronization therapy (CRT) in the presence of left bundle branch block and heart failure (HF) [31]. Other types of wide QRS HF have enjoyed less success, notably right bundle branch block. The adverse effects of left bundle branch block emerge with age/disease as compromised cardiac function [32]. When cardiac reserve is reduced, minor electrical aberrations have a much greater impact on function. The world of pacing has and is undergoing a revolution, but ignorance and lack of conceptual thinking is bliss. How foolish could we have been even to think that right ventricular pacing, with its abnormal ventricular depolarization, would not be detrimental to cardiac function with or without disease. It took the DAVID study, comparing atrial pacing with ventricular pacing in Defibrillator patients without AV block, to highlight this, where ventricular pacing unnecessarily resulted in reduced ventricular function [33]. This deterioration occurred relatively quickly in these patients with already damaged ventricles. We were, of course, at that time limited by the available lead technology and its link with anatomy. The right ventricular (RV) apex was the best place to achieve electrode stability without screw‐in electrodes, but this was changed by the advent of active fixation (screw‐in) leads [34]. Furthermore, we failed to conceive that interventricular dyssynchrony would result from RV pacing, and in patients requiring pacing without AV block (e.g., SND), also, the inevitability of additional atrioventricular (AV) dyssynchrony.

The MOST study [10] emphasized the problem of RV pacing and its negative inotropic burden in terms of how much the RV was paced, thus pointing out its reduction to be considered standard of care. Even then recommendations continued that RV pacing may be considered in patients with preserved systolic function. The RV outflow site was tried to minimize the deleterious effect of RV apical pacing [35]. It was also thought that the deleterious effects were confined to or greater in patients with structural heart disease [36]. Algorithms were developed to minimize ventricular pacing which were of benefit in reducing unnecessary RV pacing but were inappropriate in most patients with complete heart block requiring continuous ventricular pacing and less effective than hoped in some of those not requiring continuous pacing [37]. It has been only over the last decade or so when attempts have been made to avoid RV pacing by selecting a more physiological mode.

The mere avoidance of RV pacing is not sufficient in most instances where significant conduction disease exists. Pacing the ventricles from the left and right sides of the septum, thereby attempting to engage different sites in the conduction system, has led to left bundle area pacing. The precise site for optimal pacing may well be different in each patient depending on the state of the intrinsic conduction system. However, the concept of left bundle pacing may well be too simplistic.

Now, there is realization that the best way to achieve optimal cardiac function is to mimic nature as closely to that in the normal heart as possible. Further, in the context of disease with less cardiac reserve, electrical conduction abnormalities are greatly magnified in their effect on mechanical function as was well illustrated by the DAVID study [33]. It, therefore, took around 10 years finally to accept that RV pacing was detrimental both for optimal cardiac function but also for increased incidence of atrial fibrillation [18, 38]. Though this field has evolved, there remains far to go. While appreciation of ventricular resynchronization has taken hold, we have often ignored the importance of atrial electrical activation and have prematurely considered the AV relationship solved, simply by AV sensing and pacing.

## 6 From Right Ventricular Apical Pacing to Pacing for Heart Failure

This has been a long road which can be traced back to work from Innsbruck in the late 1980s [39]. Their cardiac transplant team had a long waiting list prompting them to examine those patients very carefully. Many were bradycardic. So, they paced them with DDD devices. Many substantially improved from which observation a conclusion was drawn that physiological pacing may be good for HF. It is probable that the good effects were twofold ‐ correction of bradycardia and improved AV synchrony. Their findings sparked widespread thought about pacing possibilities in HF. Brecker et al. [40] followed with another small series more clearly defining the mode of benefit, earlier seen in Innsbruck, was mainly AV synchrony. Then, the group in Val d'Or, Paris performed 4 chamber pacing in a single HF patient [41] with striking benefit emphasized by subsequent left ventricular lead displacement resulting in severe deterioration. Repositioning the displaced lead combined with some diuretics restored his good condition. This single patient vividly displayed the case for biventricular (at a minimum) pacing. In the late 1990s CRT was realized as a therapy for HF patients with a wide QRS [42]. Still at this stage, there was much to be understood. Which patients gained most, where to place the electrodes, when and where to pace the right and left ventricles etc. were questions requiring answers.

Patients with HF were not generally thought to benefit from pacing unless they had coincident AV block. Most were under the care of HF cardiologists who had to be convinced that they could safely refer their patients to pacemaker electrophysiologists. This proved to be quite a long process which, when completed, saw widespread use of CRT and cooperation of the two subspecialties to offer better care.

## 7 Dyssynchrony

Resynchronization can only be of benefit if there is pre‐existing dyssynchrony. The HF that is being treated is ventricular dyssynchrony which is due to electrical conduction abnormalities. It is now well understood that electrical activation sequence and timing can significantly impact cardiac performance. Any such aberration can result in dyssynchrony. Undoubtedly, the most recognized example is the dyssynchrony created by left bundle branch block. Here it is both the activation sequence and the delay in activation of the basal left ventricle (LV) resulting in septal dyssynchrony and systolic dysfunction. The impact of these abnormalities is much greater in the presence of cardiac disease with pre‐existing dysfunction. Interestingly, right bundle branch block has no perceptible impact on overall cardiac function. In addition to LV dysfunction there may be atrioventricular (AV) dyssynchrony stemming from disturbance of relative timing of atrial and ventricular activation affecting ventricular filling. AV interval abnormalities have different significance for mitral and tricuspid regurgitation. AV valvular regurgitation is controlled by papillary muscle contraction. If these muscles are activated too late regurgitation will surely result. Interatrial dyssynchrony because of interatrial conduction delay has not been well studied but it has importance. These electrical issues leading to dyssynchrony are greatly accentuated by the presence of cardiac disease. An important cardiac disease, in this context, is myocardial damage due to coronary artery disease and to aging. Here, cardiac magnetic resonance imaging is very helpful in anticipating where conduction is impaired together with its effect. Furthermore, these diseases are usually not static pointing to a need to keep resynchronization under constant review.

It should not be a surprise that those patients presenting HF with little or no QRS prolongation will not benefit from resynchronization. However, even subtle prolongations could be ameliorated by subtle adjustments in stimulation site and stimulus timing and always in concert with AV timing. Here, attention must be directed to atrial pacing in both atria or possibly at Bachmann's bundle with focus on site and timing of stimuli.

## 8 Cardiac Resynchronization Therapy

Cardiac resynchronization therapy stands as a significant advance in the treatment of HF, particularly for patients with symptomatic systolic HF caused by ventricular dyssynchrony [43, 44]. Addressing this condition, which affects a substantial portion of HF patients, CRT aims to provide the failing heart with a mechanical advantage, leading to notable improvements in symptoms and mortality rates. Upon activation, CRT promptly mitigates all three types of cardiac mechanical dyssynchrony, enhancing cardiac output through coordinated ventricular contraction. Beyond immediate hemodynamic benefits, CRT initiates a process of reverse remodeling over several months, characterized by structural and functional improvements in the heart [44]. This long‐term transformation typically involves a reduction in LV size and enhancement of LV function judged by ejection fraction, consistently observed in CRT‐treated patients with symptomatic HF and prolonged QRS duration. While CRT employing biventricular pacing (BVP) has demonstrated success in some HF patients, including those requiring anti‐bradycardia pacing with normal ejection fraction, its implementation often necessitates more complex implantation procedures and may present challenges such as elevated coronary sinus lead thresholds and phrenic nerve capture issues [45, 46].

CRT has in most cases employed RV pacing, usually at the apex, and the lateral epicardial surface of the LV via the coronary sinus. Coronary sinus lead placement was a greater challenge demanding specific equipment and more skill. Eventually, this became routine too. With BVP, the timing of left atrial activation was mostly ignored even though, if inappropriate, it could vastly diminish the benefit of pacing both ventricles [47]. Furthermore, the timing of stimulation of the two ventricles was sometimes also overlooked. On the same theme, the site of ventricular stimulation could play a major part in success or failure of treatment of HF. It must be borne in mind that epicardial stimulation of the LV as occurs from coronary sinus leads yields activation in the opposite direction to normal.

The advent of coronary sinus quadripolar lead has enabled both basal and multisite LV pacing [48]. RV pacing in BVP may still be harmful as evidenced by the superiority of LV pacing alone in some instances [48]. CRT has also been shown to improve mortality in a large clinical trial [49].

A new phrase appeared in the clinical dictionary “nonresponder,” one that had not been applied in drug treatment. The nonresponder group formed about 30% of those treated. Some of these unfortunate patients suffered from malpositioning or maltiming of stimulation. It should be added that use of the coronary sinus approach offered limited access to ideal stimulation sites because of the individual anatomy of the heart's venous system. We had embarked on a very complex therapeutic exercise that required the deepest electrophysiological thought, which sadly has not always been given to the problem.

## 9 His Bundle Pacing (HBP)

HBP presents an alternative to traditional RV and BVP techniques, aiming to preserve physiological ventricular activation through the native His‐Purkinje system. Its clinical advent was very slow, in the United States there was little interest, despite the good efforts by Deshmukh [50], and in Europe, Zanon was almost a single‐handed protagonist [51]. His bundle pacing (HBP) became a reality under the guidance and skill of Vijayaraman and team [52] who again were initially lone protagonists, soon applying the technique to HF indications [53]. The benefits of reproducing quasi‐normal ventricular activation were readily evident. Surprisingly, pacing the His bundle overcame conduction blocks below it although this required increased energy delivery in some cases [54]. Another potential advantage of HBP over RV pacing lies in the reduction of functional tricuspid regurgitation, particularly evident in proximal His bundle implants where the lead position is atrial to the tricuspid valve [55]. Although conclusive evidence of this mechanical benefit remains to be established, it is not expected when combining His pacing with transvalvular lead systems such as implantable defibrillator leads or with concurrent RV pacing and distal His/conducting system implants. The conceptual advantages of HBP over RV pacing, including reduced QRS duration and improved ventricular activation patterns, are increasingly acknowledged [56]. Nonetheless, despite its conceptual benefits, HBP comes with several limitations. These include prolonged fluoroscopy times, elevated pacing thresholds related to the sparse cardiac muscle in that region in addition to the greater energy (at para‐Hisian sites) required to achieve narrowing of the paced QRS complex. The increased pacemaker outputs lead to much earlier battery depletion in comparison with RV pacing. There are also more frequent lead dislodgements [57].

## 10 Left Bundle Branch (LBB)/Left Bundle Branch Area Pacing (LBBAP)

LBBAP has emerged as a promising alternative for achieving physiological pacing, offering improved stability and long‐term pacing thresholds [58, 59]. This innovative pacing approach involves capturing the LBB and employing mechanisms to narrow the QRS as in HBP [59] via a transventricular septal approach. Initial clinical studies have demonstrated the feasibility and safety of LBBAP, with rare complications and high success rates observed. LBBAP offers improved electrical synchrony of the left ventricle, characterized by narrow or narrower paced QRS duration, more normal depolarization patterns, rapid synchronized left ventricular activation, and correction of left bundle branch block [58]. As a result, LBBAP holds potential as an alternative pacing modality for both traditional RV apical pacing and cardiac resynchronization therapy, either independently or in combination with HBP [59, 60, 61]. As a result of a consistent ability to engage the left bundle the term left bundle area pacing has evolved. In this context, it must be remembered that there may be distal His bundle disease which could still prejudice conduction [62]. A major issue with the technique is that the interventricular septum is thick and fibrotic, and the depth attained with the pacing lead with variable sheath support either having inadequate penetration or over‐penetration into the left side of the interventricular septum [63]. Furthermore, validation through randomized clinical trials is required to confirm its safety and efficacy before widespread adoption can be recommended. An early report of medium‐term follow‐up suggests that at ~9 months loss of quality LV function is unusual and attributable to potentially reversible technical problems of adequacy of pacing or AF [64].

So far, leadless pacing has made only an AV synchrony contribution to physiological pacing. Two different dual chamber systems are now available [65, 66, 67], but they are unable yet to perform HBP or LBBAP. In the future multiple precisely placed devices, with inter‐device communication, will be required to achieve leadless physiological pacing. Consideration has been given to the siting for a leadless device in the right atrium on an anatomical basis, but this has not included electrophysiological appropriateness [68].

## 11 Imaging and Physiological Pacing

As a result of individual differences in the conduction system true physiological pacing could be best determined by imaging at the time of site selection as well as in assessing its long‐term consequences. Understanding that the acute impact of these pacing modes and sites may not be readily apparent from standard techniques. Only using sophisticated assessment of long‐term pacing may these differences become apparent. For example, this was how nonresponders to CRT were revealed by analysis of both V‐V and A‐V timing, using echocardiography guided pacemaker programming [69, 70]. Left bundle area pacing can also result in paradoxical septal motion which will be readily appreciated by imaging [62].

## 12 Atrioventricular Junctional Ablation–Could This Ever Be Considered Physiological?

The instant answer must be ‘no’ as it is a procedure which inflicts damage to part of the cardiac conduction system. However, in the context of permanent atrial fibrillation a controlled heart rate is considered a highly desirable step toward a more physiological state. Thus, atrioventricular junctional (AVJ) ablation, damaging though it is, achieves that improved physiological state when combined with a form of ventricular pacing. Today, this is His bundle pacing which delivers quasi‐physiological activation of the ventricles. This may protect the patient from deteriorating ventricular function and, also, the existing small sudden death rate early after ablation. However, if ventricular pacing could achieve a normal activation pattern of the right and left ventricle this may still have a role in permanent AF where rate control is not feasible with drug therapy alone. Thus, there is a possibility of an aspect of physiologic pacing to be applied after AVJ.

## 13 Present and Future of Physiological Pacing

Now, it is necessary to regard cardiac pacing as holistic therapy taking all aspects of the patient's cardiac problems into account. It is hoped that physiological pacing may evolve into complete reproduction of what happens in the normal heart. Achieving this goal must imply consideration of ventricular diastole as well as systole and, also, precision of timing of activation of both atria and ventricles. The role for leadless pacing here is not yet identified but without doubt this must incorporate the concept of physiological pacing and what we have learnt from using pacing leads over the last 40 years should be fully employed with leadless pacing.

Physiological pacing could be summarized very briefly; in the presence of normal conduction and activation of the atria and ventricles, pacing replicates this without distortion, and In the presence of abnormal atrial and ventricular activation, pacing rectifies the abnormalities (Central Illustration 1).

**Central Illustration 1:**
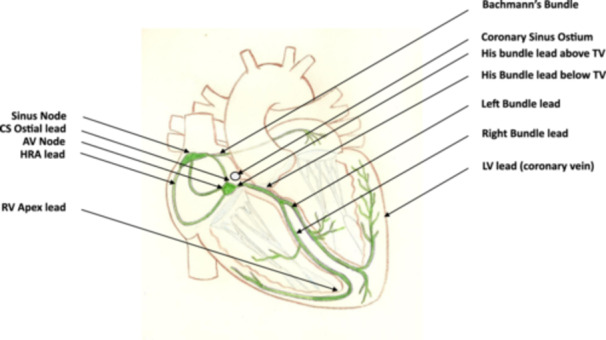
The figure shows a diagrammatic heart with its conduction system in green on which is labelled the various sites at which physiological cardiac stimulation can be delivered via leads. AV, atrioventricular; CS, coronary sinus; HRA, high right atrial; LV, left ventricular; RV, right ventricular; Tv, tricuspid valve.

## Ethics Statement

The authors have nothing to report.

## Conflicts of Interest

The authors declare no conflicts of interest.

## Data Availability

The data that support the findings of this study are openly available in references cited.
